# Extraintestinal Salmonellosis in the Immunocompromised: An Unusual Case of Pyomyositis

**DOI:** 10.1155/2017/5030961

**Published:** 2017-09-11

**Authors:** Veeraraghavan Meyyur Aravamudan, Phang Kee Fong, Pavel Singh, Jong Sze Chin, Yang Shiyao Sam, P. A. Tambyah

**Affiliations:** ^1^Department of Medicine, National University Hospital, 5 Lower Kent Ridge Road, Singapore 119074; ^2^Department of Diagnostic Imaging, National University Hospital, 5 Lower Kent Ridge Road, Singapore 119074; ^3^Division of Infectious Diseases, National University Hospital, Singapore

## Abstract

Salmonella infection can cause a wide range of presentations, predominantly gastrointestinal but occasionally with cardiovascular or other extraintestinal manifestations. The diagnosis of extraintestinal salmonellosis requires a high degree of clinical suspicion and should be considered in patients with deep-seated abscesses especially if they are immunocompromised. We present a case of salmonella causing gastroenteritis complicated by an intramuscular abscess of the left leg. With prompt recognition and multidisciplinary management, the patient recovered with no serious sequela.

## 1. Introduction

Salmonella infection can cause a wide range of presentations especially in immunocompromised hosts. In this case, we describe a 55-year-old Chinese Singaporean man, who presented with acute onset of nonbilious and nonbloody vomiting with diarrhoea and left leg pain. He was found to have gastroenteritis caused by* Salmonella* Group C complicated by an intramuscular abscess of his left leg. We reviewed the literature in the context of this unusual presentation.

## 2. Case Report

A 55-year-old Chinese Singaporean man with a background of Polycythemia Rubra Vera (PRV), currently on treatment with hydroxyurea and anagrelide, diabetes mellitus, and hypertension, was admitted with a four-day history of nonbilious, nonbloody vomiting accompanied with nonbloody diarrhea. This was associated with fever and also left leg pain and swelling. He had no other infective symptoms including cough, sputum production, or dysuria.

Physical examination revealed pyrexia (temperature: 38.9 degrees Celsius), blood pressure of 140/60 mmHg, and pulse rate of 80/min. His left calf was erythematous, swollen, warm, and tender. His abdomen was soft and nontender with no bruits. No pitting oedema was present. Routine blood investigations showed normal inflammatory markers and biochemistry (Table 1). Ultrasound of the left lower limbs showed thrombosis of the left posterior tibial vein. Blood and stool cultures on admission grew Group C* Salmonella*.

He was started on intravenous ceftriaxone and subcutaneous low molecular weight heparin (LWMH) for deep venous thrombosis.

However, he had persistent fever and became hypotensive on the 10th hospital day. Repeat blood cultures grew Group C* Salmonella*, but repeat stool culture grew Group B* Salmonella*. Transthoracic echocardiogram showed no evidence of vegetation. Computed tomography (CT) aortography did not reveal aortitis.

**Figure 1:**
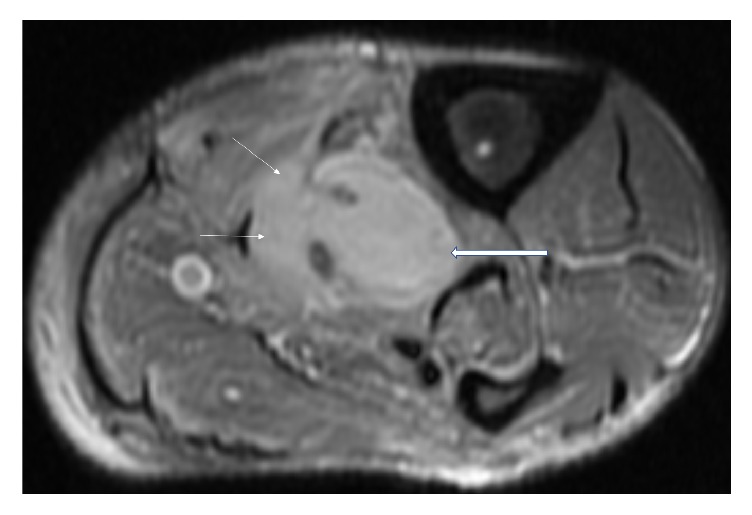
Axial, T2 weighted, fat saturated, STIR MR image showing loculated fluid signal within the tibialis posterior muscle (thick white arrows), suggestive of a collection. Also noted are diffuse T2 hyperintense signal changes in the surrounding musculature (thin white arrows), compatible with myositis.

**Figure 2:**
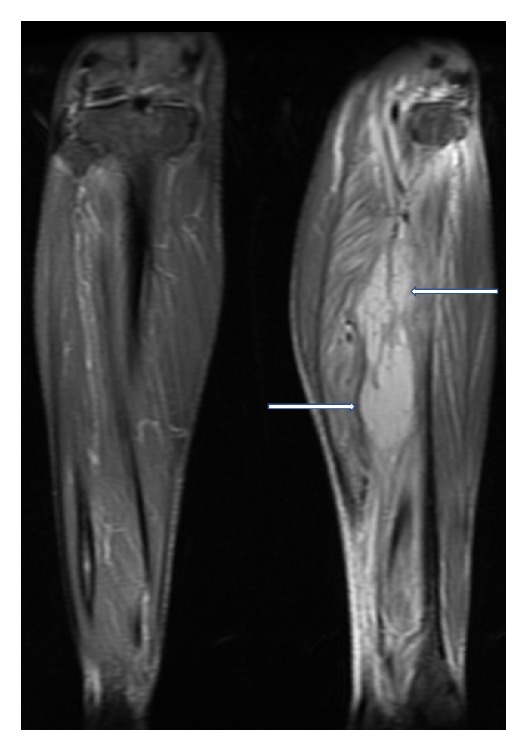
Coronal, T2 weighted, fat saturated, STIR MR image showing loculated fluid signal within the tibialis posterior muscle (thick white arrows), suggestive of a collection. Also noted are diffuse T2 hyperintense signal changes in the surrounding musculature (thin white arrows), compatible with myositis.

**Figure 3:**
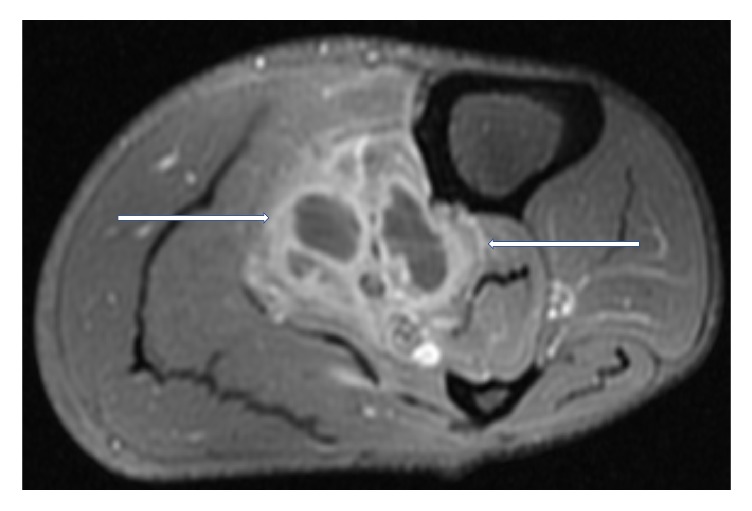
T2 weighted, fat saturated, postcontrast, axial MR image of left calf showing a multiloculated fluid collection with associated enhancing rim and septations (white arrows) within the tibialis posterior muscle, compatible with an intramuscular abscess.

**Figure 4:**
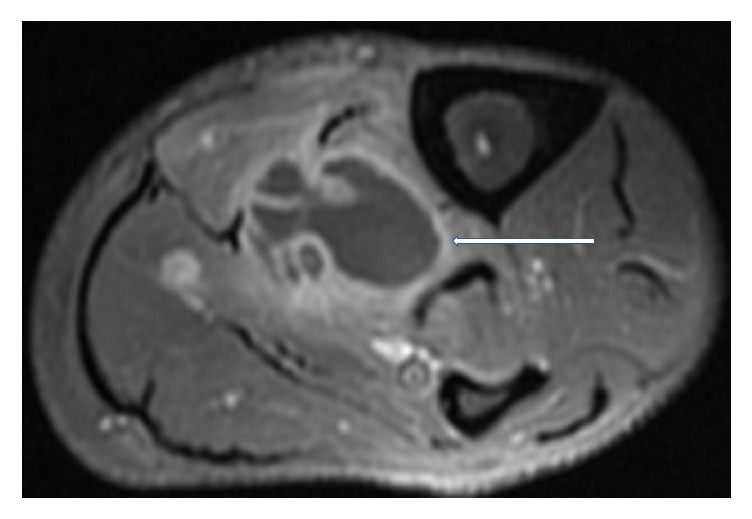
T2 weighted, fat saturated, postcontrast, axial MR image of left calf at another location, again showing a multiloculated fluid collection with associated enhancing rim and septations (white arrows) within the tibialis posterior muscle, compatible with an intramuscular abscess.

**Figure 5:**
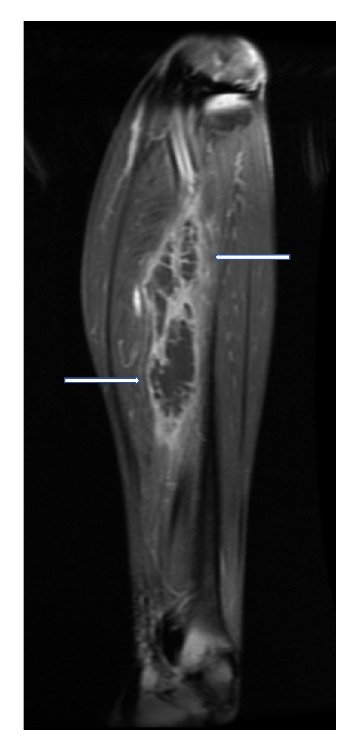
T2 weighted, fat saturated, postcontrast, coronal MR image of left calf showing a multiloculated fluid collection with associated enhancing rim and septations (white arrows) within the tibialis posterior muscle, compatible with an intramuscular abscess.

Magnetic Resonance Imaging (MRI) of the left lower limb was performed. It showed a multiloculated collection with enhancing rim and septa centered within the tibialis posterior muscle, measuring approximately 13.9 × 4.4 × 4 cm with adjacent myositis and fasciitis (Figures 1, 2, 3, 4, and 5).

He was referred to orthopaedics for incision and drainage. Intraoperatively, a multiloculated intramuscular abscess in posterior tibialis muscle was observed; 15 mls of frank pus was drained. Two drains were inserted, with the inferior drain at the residual space of the posterior tibialis muscle and superior drain into the space between the posterior tibialis and soleus. Intraoperative pus culture grew Group B* Salmonella*.

His condition improved dramatically following the drainage with resolution of pyrexia and hypotension. IV ceftriaxone was continued for a total duration of six weeks with oral metronidazole. LWMH was administered for six weeks as well. He remained well six months after discharge.

## 3. Discussion

Typhoid fever, caused by* Salmonella enterica *serovar Typhi, is an important disease in many developing countries. It is estimated that there are approximately 22 million typhoid cases and ~200,000 deaths per year worldwide [1].

Enteric fever is caused by* Salmonella* Typhi or Paratyphi. In contrast, focal infections such as osteomyelitis and endovascular infections tend to be caused by nontyphoidal salmonellae (NTS). While NTS commonly cause endovascular infections with mycotic aneurysm of aorta as the most common site [2, 3], myositis and other soft tissue infections have been previously described [4, 5]. The type of infection depends on both host factors and, to a lesser degree, on the serotype of salmonellae [6].

Transmission usually occurs by the oral-faecal route [7] via consuming contaminated drinking water and food sources, such as poultry, eggs, dairy products, vegetables, and fruits. Our patient did not give a clear history of dietary indiscretion but half-boiled eggs are commonly consumed in Singapore and other parts of Southeast Asia [8, 9].

In microbiology laboratories, salmonellae are now rapidly identified using automated systems [10]. Interestingly, two different salmonellae strains were found in our patient and this may reflect his underlying heavily immunocompromised state [11].

Salmonella infection of the thigh has previously been reported but in association with a pseudoaneurysm of the femoral artery [4].

Current literature regarding epidemiology of extraintestinal salmonellosis in immunocompromised patients remains scarce. A Malaysian study on NTS bacteraemia found that 55 out of 56000 blood cultures collected over four years grew NTS. An extraintestinal focus was found in 30% of cases, most commonly the lung and soft tissues (7.3% each). The study also described higher mortality in immunocompromised patients (30.6% versus 5.3% in immunocompetent patients) [12].

Atherosclerosis and HIV infections are the most common predisposing factors for systemic salmonella infections [13, 14], although recently the emergence of defects or autoantibodies directed against the interferon gamma pathway has been shown to be a major underlying cause of systemic salmonella infections, particularly in Southeast Asia [15].

Warning signs of extraintestinal infection would include localized tenderness in the musculoskeletal system, pleuritic pain suggesting a pleural pathology, chest pain, or embolic phenomena suggesting a mycotic aneurysm.

In our patient, despite adequate treatment of his salmonella bloodstream infection and his deep vein thrombosis with ceftriaxone and LWMH, he became hypotensive and it was only with drainage of his intramuscular abscess that his condition began to improve.

It is important that physicians be alert for timely diagnosis of extraintestinal salmonella infections, especially in immunocompromised patients with the risk of complications which may be potentially fatal [16].

## 4. Learning Value

The diagnosis of extraintestinal salmonellosis necessitates a high degree of clinical suspicion. The majority of individuals with immunocompromised immune systems present with extraintestinal infections. A multidisciplinary approach is needed to treat immunocompromised patients with extraintestinal salmonella infections and abscesses.

## Figures and Tables

**(a):** 

Test	Results	Unit	Reference interval
White blood cell	7	×10^9^/L	3.40–9.60
Red blood cells	3.06	×10^12^/L	3.70–9.60
Haemoglobin	10.9	g/dL	10.9–15.1
Mean cell volume	105.6	fL	80.0–95.0
Mean corpuscular haemoglobin	35.6	pg	27.0–33.0
Mean corpuscular haemoglobin Concentration	33.7	g/dL	32.0–36.0
Haematocrit	32.3	%	32.7–44.4
Platelets	941	×10^9^/L	132–372
Mean platelet volume	11.7	fL	8.7–12.2
Red cell distribution width	17.8	%	11.4–14.8
Sodium	135	mmol/L	135–145
Potassium	4.0	mmol/L	3.5–5.0
Chloride	99	mmol/L	95–110
Carbon dioxide	23	mmol/L	22–31
Creatinine	113	umol/L	50–90
Urea	7.3	mmol/L	2.0–6.5
Glucose	8.6	mmol/L	4.0–7.8
Albumin	38	g/L	38–48
Bilirubin, total	2	umol/L	5–30
Bilirubin, conjugated	1	umol/L	0–5
Aspartate aminotransferase	40	U/L	10–50
Alanine aminotransferase	35	U/L	10–70
Alkaline phosphatase	99	U/L	40–130
Lactate dehydrogenase	693	U/L	250–580
Calcium, total	2.30	mmol/L	2.15–2.55
C-reactive protein	125	mg/L	0–10

**(b):** 

Blood culture	Drugs	Susceptibility	MIC
Blood culture grew Salmonella Group C	Ampicillin	Sensitive	≤2 mg/L
	Ceftriaxone	Sensitive	
	Ciprofloxacin	Resistant	0.500 mg/L
	Cotrimoxazole	Sensitive	40.00 mg/L

**(c):** 

Stool culture	Drugs	Susceptibility	MIC
Stool culture grew Salmonella Group C	Ampicillin	Sensitive	≤2 mg/L
	Ceftriaxone	Sensitive	
	Ciprofloxacin	Resistant	0.380 mg/L
	Cotrimoxazole	Sensitive	≤20 mg/L

**(d):** 

Stool culture	Drugs	Susceptibility	MIC
Stool culture grew Salmonella Group B	Ampicillin	Resistant	≥32 mg/L
	Ceftriaxone	Resistant	>256.000
	Ciprofloxacin	Sensitive	0.008
	Cotrimoxazole	Sensitive	≤20 mg/L
	Azithromycin	sensitive	4.000
